# Course and Outcome of Children with Convulsive Status Epilepticus Admitted to a Pediatric Intensive Care Unit

**DOI:** 10.7759/cureus.4471

**Published:** 2019-04-16

**Authors:** Madhuradhar Chegondi, Mary M Garland, Prithvi Sendi, Anuj R Jayakar, Balagangadhar R Totapally

**Affiliations:** 1 Pediatrics, University of Iowa Stead Family Children's Hospital, Iowa, USA; 2 Surgery, Herbert Wertheim College of Medicine, Miami, USA; 3 Pediatrics, Nicklaus Children’s Hospital, Miami, USA

**Keywords:** children, status epilepticus, outcome, pediatric intensive care unit

## Abstract

Introduction

The objective of this study was to describe the course and the outcomes of children with convulsive status epilepticus and to evaluate the differences between two groups of children with new-onset seizures and known seizure disorders.

Methods

This is a retrospective, single-center study. Children with convulsive status epilepticus admitted to our tertiary care pediatric intensive care unit were included in the study. Medical records were reviewed to obtain the demographic- and seizure-related variables.

Results

Among 139 children with status epilepticus, 69.7% (*n* = 99) had a known seizure disorder. Focal seizures were present in 23.9% of children, and 34.6% required intubation; there was an overall mortality rate of 1.2%. The children with new-onset seizures were younger and received electroencephalography (EEG) and neuroimaging more often compared to children with known seizure disorders (*p* < 0.05). However, an abnormal EEG was more common among children with known seizure disorders (*p* < 0.001).

Conclusions

Sub-therapeutic anti-epileptic drugs levels were common among children with known seizure disorders presenting with status epilepticus. Gender, race, insurance status, type of seizures, intubation requirement, lengths of stay, and mortality were not significantly different between the two groups.

## Introduction

Status epilepticus (SE) is the most common pediatric neurological emergency in children [1]. SE is defined as a seizure or cluster of seizures lasting more than 30 minutes [2]. In clinical practice, however, it has been suggested that any seizure or series of seizures lasting greater than five minutes could be considered status epilepticus because seizures rarely last this long and most children with seizures lasting for more than five minutes require pharmacotherapy to control seizures [2-3]. Recently, Tinka et al. proposed a 10-minute duration to define focal SE and 60 minutes duration of focal seizure for possible long-term consequences [4]. A substantial amount of experimental data supports the idea that prolonged seizures cause neuronal injury. Thus, prompt administration of treatment is considered critical [5]. The overall incidence of SE is reported as 10-58 per 100,000 per year in children up to 19 years of age [6]. Up to one-third of children with underlying seizure disorders experience SE [6]. In children, the incidence of SE is higher compared to adults; however, the mortality with SE in older adult patients is higher, up to 20%, and in children under the age of 10 years, it can be as low as 2.6% [7]. In 2014, the total cost of inpatient admissions for SE was 4 billion dollars [7]. While the associated mortality can be high, the rate of recurrence has been reported as low as 17% [6].

Early diagnosis and treatment significantly reduce mortality and are the key steps treating SE [8]. Following initial supportive management (airway, breathing, circulation, intravenous [IV] access), seizure control by IV lorazepam as the first-line treatment is recommended [9-10]. Alternatives for IV lorazepam include IV midazolam or IV diazepam. If there is no IV access, midazolam can be given via buccal, intranasal, or intramuscular administration. Diazepam can also be given rectally [9-10]. First-line medications control 80% of SE when given within the first 30 minutes of seizing [9]. If the patient continues to seize, an additional treatment must be administered quickly [9]. Most experts recommend fosphenytoin as second-line therapy, although there is support for the use of alternative anti-epileptic drugs (AEDs) such as phenobarbital, valproic acid, or levetiracetam [8,11]. While most causes of SE are due to epilepsy, children with atypical febrile seizures, cerebral hypoxia, and inborn errors of metabolism can also present as SE [12-13]. A 2011 Serbian study found that in general, the prognosis for children with SE is favorable; however, morbidity and mortality were highly associated with underlying etiology as well as prior neurological abnormalities [14].

The objectives of this retrospective chart review study were to describe the course and the outcomes of children with SE who are admitted to a tertiary care pediatric intensive care unit (PICU) in the United States and to evaluate the differences between children with new-onset seizure and known seizure disorder.

## Materials and methods

This is a retrospective, single-center, descriptive study. After Institutional Review Board approval, we obtained the list of children admitted to Nicklaus Children’s Hospital (formerly Miami Children’s Hospital) PICU with SE between 2005 to 2010. SE is defined as a continuous seizure lasting more than five minutes and/or multiple seizures between which consciousness is not regained for at least 30 minutes. Children aged over one month to 18 years were included in the study. Neonates, children with SE with undocumented duration of seizures, and children with undocumented antiepileptic medication administration and resolved seizures with back-to-baseline sensorium before hospital admission were excluded. We also excluded those who developed convulsive or non-convulsive SE after admission. Patients who were discharged from the hospital and readmitted to the PICU with SE were treated as separate cases. Patients who were diagnosed with SE more than once (transferred to the pediatric floor and admitted back to PICU with SE) without being discharged from the hospital were treated as one case.

Patient charts were reviewed for demographics, type of seizure (focal versus generalized), new versus known seizure disorder, length of seizure disorder, AEDs before admission, rescue AEDs used, AED levels at the time of admission (therapeutic or sub-therapeutic), interventions, etiology, length of PICU and hospital stays, and mortality. Gender and race were calculated based on total children and all other variables calculated based on total episodes of SE. Continuous data are presented as median and inter-quartile range (IQR), and categorical data are presented as a percentage. Data in two categories, new-onset versus known seizure disorder, were compared using the Mann-Whitney U test (continuous data) and Chi-square/Fisher’s exact test (categorical data). A* p*-value of less than 0.05 was considered significant.

## Results

The chart review yielded a total of 139 children with SE admitted to the PICU over the course of five years. Among the 139 children, 162 episodes of SE were identified. About 69% (*n* = 96) of children had a known seizure disorder, and among them 55.3% were male and 50.5% were Hispanic. Children with a new-onset seizure disorder were 31% (*n* = 43), among them 51.2% were females and 41.9% were Hispanic. The percent of children with repeat admissions due to recurrent SE was 10% (*n* = 14). At the time of admission in children with a known seizure disorder, 11.5% were not on any seizure medication and 55% were taking two or more AEDs. AED level data were available in 84.8% of children, of which 50.4% children had sub-therapeutic levels of AED. Focal seizures were present in 23.9% of SE episodes.

Administration of lorazepam was the most common rescue therapy in both known and new-onset seizures (58.8% vs. 57.1%; *p* = not significant [NS]). The most common second-line AED therapy used was fosphenytoin among known and new-onset seizures (53.8% vs. 57.2%; *p* = NS). Continuous infusion of midazolam was used, 17.7% of the time in known seizure disorder patients and 9.5% of the time in new-onset seizure children. Pentobarbital infusion was used in 8.3%, and both midazolam and pentobarbital infusions were used in 4.1% of children with a known seizure disorder and none in children with new-onset seizures. Endotracheal intubation was required in 34.6% of children, with no significant difference between known and new-onset SE groups (35.3% vs. 32.6%).

Gender, race, insurance status, type of seizures, intubation requirement, and mortality were not significantly different between the two groups (Table 1). EEG was done in 74% of all children admitted with SE. Of them, 69% had abnormal findings. Among children with new-onset SE, 93% had EEG evaluations. The children with new-onset seizures were younger and received EEG and neuroimaging more often compared to the children with known seizure disorder (*p* < 0.05; Table 1). An abnormal EEG was more common among children with known seizure disorders (*p* < 0.001). Lengths of stay were not different between the two groups. 

**Table 1:** Demographic and clinical characteristics of the children presenting with status epilepticus ^1^status epilepticus; ^2^not significant; ^3^interquartile range; ^4^electroencephalogram; ^5^pediatric intensive care unit

Variable	All episodes of SE^1^ (n = 162)	New-onset seizure disorder, 43 out of total 139 children (31%)	Known seizure disorder, 96 out of total 139 children (69%)	p-value
Male (%)	55.3	48.8	54.2	NS^2^
Hispanic (%)	47.5	41.9	50.5	NS
Age (years) [median (IQR^3^)]	4 (1–10)	3 (1–8)	4 (2–11)	<0.05
Weight (kg) [median (IQR)]	18 (12–40)	15 (10–36)	19 (12–42)	NS
Insured (%)	92.6	86	95	NS
Focal seizures (%)	23.9	27.9	21.8	NS
Intubated (%)	34.6	32.6	35.3	NS
Inotropic support	0.6	0	0.8	NS
EEG^4^ during admission (%)	74.5	93	67.8	<0.001
Abnormal EEG (%)	69.1	53.5	74.8	<0.05
Imaging during admission (%)	70.3	95.3	61.3	<0.0001
Abnormal imaging (%)	45.7	65.1	38.7	<0.0001
PICU^5^ Length of stay (days) [median (IQR)]	3 (2–4)	2 (2–3)	3 (2–5)	NS
Hospital Length of stay (days) [median (IQR)]	6 (4–11)	5 (4–8)	6 (4–13)	NS
Mortality (%)	1.2	0	1.68	NS

The overall mortality rate was 1.2%. Two children with known seizure disorder died. Among them, one child had a hypoxic-ischemic brain injury with severe comorbidities, and another child died due to refractory infantile epilepsy. Demographic, course, and outcome data are presented in Table 1. Idiopathic epilepsy in children with a known seizure disorder and new-onset seizures was 36.4% and 37.2%, respectively. Central nervous system (CNS) infection was 5.2% in children with known seizure disorder and 11.6% in children in the new-onset seizure group. Other etiologies include genetic or metabolic, febrile seizure, and stroke. Etiological data of both groups are presented in Table 2.

**Table 2:** Etiology of status epilepticus in children in new-onset and known seizure disorder groups

Etiology	New-onset seizure group (n = 43)	Known seizure disorder group (n = 96)
Idiopathic	16	35
Encephalitis/meningitis	5	5
Febrile seizure	9	6
Intracranial mass	5	5
Epilepsy with fever	4	27
Genetic/metabolic	2	18
Stroke	2	0

## Discussion

Demographics

Our study included 139 children; there was an equal distribution of male and female children in the new-onset SE group, and 54.2% of children were male in the known seizure disorder group. AED levels were sub-therapeutic in children with a known seizure disorder in the majority of children with breakthrough seizures. About one-third of children with SE were intubated in our cohort. In a study of 302 children admitted with SE, gender distribution was not significantly different as in our study [14]. Other studies have also failed to demonstrate a stronger prevalence of SE in either sex [15]. Hispanic patients made up roughly half of our total sample size (56.3%). Our institution’s results were consistent with the ethnic distribution in the South Florida region [16]. The median age for our patient population was four years for all patients with seizures and three years of age for new-onset seizure presentations. This is higher than the age reported by Lacroix et al. during their 10-year study of all SE admissions to a PICU [12]. Lacroix et al. found that out of 147 children with SE, 51% occurred before two years of age with a median age of occurrence being one year [12]. By contrast, in a study published by Dragoumi et al., the median age at onset of epilepsy was 6.5 years of age [17].

Types of seizures

The distinction between generalized and focal seizures is essential because the prognosis and the response to treatment can often be predicted based on the type of seizures [8]. Our study found that 76.2% of patients presented with generalized seizures and 23.8% with focal seizures, similar to the study conducted by Lacroix et al. [12]. According to the 2006 recommendations from the American Academy of Neurology, an EEG monitoring should be started on any child presenting in SE to help differentiate a focal from a generalized epileptiform abnormality as well as to help rule out a pseudoseizure [6]. EEG monitoring in children with convulsive SE also helps to predict the outcome: EEGs with periodic lateralized epileptiform discharges; burst suppression patterns suggest poor outcome [14]. In addition to various clinical features and underlying etiology of SE in children with SE, the EEG pattern determines the ability of a maintenance AED regimen’s successful control of seizures [18]. About 75% of our patients had EEG monitoring, and among them 70% EEGs were abnormal. Previous studies reported 33% to 43% of EEGs of children with convulsive SE with epileptiform abnormalities [6,19]. About one-third of the patients in our study were intubated. Lacroix et al. found that 58% of the patients in their study were intubated, most commonly for airway protection, followed by respiratory arrest or secondary to sedative medication such as diazepam or phenobarbital [12]. While our study did not differentiate the reason for the need for intubation (airway protection vs. respiratory insufficiency), patients with new-onset seizures were just as likely to be intubated as patients with a known seizure disorder.

Etiology

In the previous studies, the incidence of a known seizure disorder in children admitted to the PICU with SE varied from 34% to 53.3% [11-12]. In our study, 36.7% of all children with SE had no etiology found, 10.8% had febrile seizures, and the remaining children had meningitis, encephalitis, or space-occupying lesions. Infective etiologies comprise 7.2% in our study, similar to a previous systemic review, reporting 1% to 12% of patients with SE having infectious causes in children living in countries in the developed world [20]. The American Academy of Neurology practice parameter addressing the diagnostic assessment of convulsive SE reports multiple causes of SE in children and subtherapeutic seizure being the most common etiology of convulsive SE [6].

Imaging

Neuroimaging within 60 minutes of the onset of SE recommended per neurocritical care guidelines [18]. However, stabilizing ABCs (airway, breathing, and circulation) and controlling SE take priority [6]. Among our patients, 37.7% had computed tomography (CT) imaging of the brain, 24.7% had magnetic resonance imaging (MRI) brain, and 1.2% had both CT and MRI of the brain. Abnormal findings on imaging were noted in 45.7% of patients. Our findings were similar to that reported in a previous report [21]. The International League Against Epilepsy (ILAE) Subcommittee for Pediatric Neuroimaging reviewed imaging in children with recent onset seizures and found that 50% of children with focal new-onset seizures had abnormal imaging studies [21]. In a prospective study in children with new-onset seizures presenting as SE, Singh et al. reported 20% and 58% CT and MRI brain imaging abnormalities, respectively [22].

Antiepileptic drugs (AEDs)

Even though the most common rescue therapy in our study was the administration of lorazepam, other rescue therapies include administration of diazepam, midazolam, or phenobarbital. Previous studies demonstrated similar efficacy and no preference for rescue medications among lorazepam, midazolam, and diazepam [11,23]. Fosphenytoin was used in the majority of our patients as second-line therapy. Recommended second-line therapies include fosphenytoin, valproic acid, and levetiracetam, and there is no clear evidence to suggest superior efficacy among them [11, 18]. In children with refractory convulsive SE, therapies such as continuous midazolam, pentobarbital, and propofol infusions should be considered [11]. Tasker et al. in their intention-to-treat analysis reported that midazolam infusion was first-line therapy in 78% of children with refractory SE and pentobarbital infusion was used 82% of children who failed the midazolam infusion [24]. Subtherapeutic AED level is one of the most common causes of breakthrough seizures in a child with epilepsy [6]. AED levels should be considered in all children with known seizure disorder presenting with SE [6]. In our study, among the children in the known seizure disorder group, AED levels were available in 84.8% of children, of which 50.4% of children had sub-therapeutic levels of AEDs. The American Academy of Neurology practice parameter review reported that 32% of children with SE had subtherapeutic levels of AEDs [6]. Non-adherence to AEDs is a significant factor for poor outcome in children with epilepsy regarding seizure recurrence and poor health-related quality of life [25]. In another prospective study, Modi et al. showed a strong correlation of early nonadherence to AEDs with a lower rate of long-term seizure control [26]. Therapeutic levels of AEDs help in drug adherence monitoring and in optimizing the AED regimen without resulting in drug toxicity [27].

Outcome

In our study, the overall mortality was 1.2%, which includes two patients in the known seizure disorder group. In previous studies, the mortality rate in children with convulsive SE was 3-6% [12,28]. Also, in our study, PICU length of stay and overall hospital length of stay were similar to previous studies [12]. However, in a retrospective study, in children with acute encephalitis who presented with refractory SE, the PICU length of stay was longer and these patients had a poor neurological outcome [29]. Also, in children with refractory SE, requiring pentobarbital infusion had a longer length of hospital stay [30].

Limitations

We acknowledge the limitations of our study. First, it is a single-center, retrospective analysis. Second, the course of SE was based on chart review, and the timing of the management sequence may not reflect the actual time of interventions. Finally, variation in SE management protocols in different centers may influence results in those centers, and caution should be taken when generalizing single-center study results. 

## Conclusions

While children with new-onset SE group seizures were younger and received different diagnostic evaluation compared to SE in children with a known seizure disorder, there was no difference in outcomes between the known and new-onset SE groups as far as intubation, ventilation, PICU and hospitals stays, or mortality. Sub-therapeutic AED levels are common among children with known seizure disorder presenting with SE, a potential area for future intervention.
